# Transparency of Outcome Reporting and Trial Registration of Randomized Controlled Trials Published in the *Journal of Consulting and Clinical Psychology*

**DOI:** 10.1371/journal.pone.0142894

**Published:** 2015-11-18

**Authors:** Marleine Azar, Kira E. Riehm, Dean McKay, Brett D. Thombs

**Affiliations:** 1 Lady Davis Institute for Medical Research, Jewish General Hospital, Montréal, Québec, Canada; 2 Department of Psychology, McGill University, Montréal, Québec, Canada; 3 Department of Psychology, Fordham University, Bronx, New York, United States of America; 4 Department of Epidemiology, Biostatistics, and Occupational Health, McGill University, Montréal, Québec, Canada; 5 Department of Psychiatry, McGill University, Montréal, Québec, Canada; 6 Department of Medicine, McGill University, Montréal, Québec, Canada; 7 Department of Educational and Counselling Psychology, McGill University, Montréal, Québec, Canada; 8 School of Nursing, McGill University, Montréal, Québec, Canada; Hunter College, UNITED STATES

## Abstract

**Background:**

Confidence that randomized controlled trial (RCT) results accurately reflect intervention effectiveness depends on proper trial conduct and the accuracy and completeness of published trial reports. The *Journal of Consulting and Clinical Psychology* (JCCP) is the primary trials journal amongst American Psychological Association (APA) journals. The objectives of this study were to review RCTs recently published in JCCP to evaluate (1) adequacy of primary outcome analysis definitions; (2) registration status; and, (3) among registered trials, adequacy of outcome registrations. Additionally, we compared results from JCCP to findings from a recent study of top psychosomatic and behavioral medicine journals.

**Methods:**

Eligible RCTs were published in JCCP in 2013–2014. For each RCT, two investigators independently extracted data on (1) adequacy of outcome analysis definitions in the published report, (2) whether the RCT was registered prior to enrolling patients, and (3) adequacy of outcome registration.

**Results:**

Of 70 RCTs reviewed, 12 (17.1%) adequately defined primary or secondary outcome analyses, whereas 58 (82.3%) had multiple primary outcome analyses without statistical adjustment or undefined outcome analyses. There were 39 (55.7%) registered trials. Only two trials registered prior to patient enrollment with a single primary outcome variable and time point of assessment. However, in one of the two trials, registered and published outcomes were discrepant. No studies were adequately registered as per Standard Protocol Items: Recommendation for Interventional Trials guidelines. Compared to psychosomatic and behavioral medicine journals, the proportion of published trials with adequate outcome analysis declarations was significantly lower in JCCP (17.1% versus 32.9%; *p* = 0.029). The proportion of registered trials in JCCP (55.7%) was comparable to behavioral medicine journals (52.6%; p = 0.709).

**Conclusions:**

The quality of published outcome analysis definitions and trial registrations in JCCP is suboptimal. Greater attention to proper trial registration and outcome analysis definition in published reports is needed.

## Introduction

Evidence-based psychological practice involves combining clinical experience and local context with evidence from systematic research in order to provide the best possible care to patients [1]. Accurately reported information from well-designed and conducted clinical trials is necessary in order to have confidence that research evidence reflects what would occur in clinical practice. Deficiencies in trial design and inaccurate reporting, on the other hand, can lead to the publication of biased estimates of treatment effect [2, 3], which limits the ability of practitioners to use evidence effectively in practice.

Randomized controlled trials (RCTs) of interventions often evaluate multiple clinical outcomes. However, one outcome analysis with a single statistical test should ideally be designated *a priori* as the trial’s primary outcome analysis, and additional outcome analyses should be clearly identified as secondary [4, 5]. Not clearly designating a single primary outcome analysis can make interpretation of results difficult when different outcome analyses give contradictory results, even when there is adjustment for multiplicity [4–6]. Additionally, studies that analyze multiple outcomes without specifying a primary outcome analysis *a priori* and without adjusting statistically for multiple analyses present an elevated risk of having at least one statistically significant outcome analysis, even when the null hypothesis of no treatment effect is true (i.e., Type I error) [4, 7, 8].

The failure to designate a primary outcome analysis before beginning a trial can also lead to biased estimates of effectiveness due to selective outcome reporting, which occurs when only analyses with p-values less than 0.05 are reported and those with p values greater than 0.05 are omitted from published trial reports [4, 9–12]. In addition to selectively reporting analyses of some outcome variables, but not others, selective analysis reporting bias can occur with a single outcome variable if investigators do not specify analytic methods ahead of time, but instead conduct multiple analyses (e.g., t-test, ANCOVA) and only publish those that are statistically significant. Similarly, if trial results are analyzed at multiple time points, or if outcome variables are analyzed using different metrics (e.g, change score, final score) or aggregation methods (e.g., mean scores, percent above a cutoff score), the number of analyses conducted and the chance of false positive findings in publications increases [9–11, 13, 14].

Guidelines for best practices for conducting and reporting results of trials of health care interventions, including psychological interventions, encourage adequate definition and reporting of outcome variables and analyses. The Consolidated Standards of Reporting Trials (CONSORT) [6, 15] were developed in 1996 in response to recommendations for improving the reporting of clinical trials [2]. The CONSORT statement includes a minimum set of items that should be included in reports of RCTs with a 25-item checklist to aid compliance [6, 15]. CONSORT urges *a priori* specification of primary and secondary outcome variables and analyses. CONSORT also emphasizes the importance of selecting a single primary outcome analysis to avoid problems that arise from having multiple primary outcome analyses. These guidelines are widely endorsed by journals that publish interventions to improve health, including mental health (for a full list, see: http://www.consort-statement.org/about-consort/endorsers). The CONSORT extension for non-pharmacologic treatments, such as surgery, rehabilitation, and psychotherapeutic and behavioral interventions, addresses reporting topics relevant to these types of trials, including difficulty in blinding, complexity of treatment interventions, and the influence of care provider expertise, for example [16, 17]. The American Psychological Association (APA) has developed an alternative tool, the Journal Article Reporting Standard (JARS) criteria [18], which are included in the author guidelines of most APA journals, including the *Journal of Consulting and Clinical Psychology* (JCCP). The JARS criteria provide standards for defining primary and secondary outcome analyses, but do not address the issue of single versus multiple primary outcome analyses [18].

In addition to trial reporting guidelines, trial registration policies have been introduced to reduce non-publication of entire trials that do not find statistically significant results in favor of treatments, as well as the selective publication of only statistically significant outcome analyses from trials with mixed results [19]. Since 2005, the International Committee of Medical Journal Editors (ICMJE) has required trial registration in public trial registries prior to patient enrolment for studies to be considered for publication. ICMJE guidelines require *a priori* specification of the primary and secondary outcomes in the trial registration [19]. Registration is not a time-consuming procedure; a quick internet search of websites of several academic medical centers found general agreement that, with all relevant information at hand, no more than one to two hours are required to register a trial online (see S1 Table). Trial registration policies consistent with the ICMJE policy have been widely adopted by biomedical journals beyond the ICMJE member journals, including psychology and psychiatry journals (see for example [20]). Based on author instructions posted online, however, no APA journal currently requires trial registration.

In addition to CONSORT and trial registration, the Standard Protocol Items: Recommendations for Interventional Trials (SPIRIT) guideline was developed to improve the content of clinical trial protocols [21]. SPIRIT includes a 33-item checklist with minimal items recommended to be included in trial protocols. SPIRIT recommendations include registering trials and specifying primary and secondary outcome analyses, including the specific outcome variable (e.g., Beck Depression Inventory score), a time point for each outcome analysis, the analysis metric (e.g., change from baseline, final value, time to event) and the method of aggregation (e.g., mean, proportion above or below a cutoff threshold) [21].

A recent study investigated primary outcome analysis definitions and trial registration in clinical trials published in 2013–2014 in four top psychosomatic and behavioral medicine journals: *Annals of Behavioral Medicine*, *Health Psychology*, *Journal of Psychosomatic Research* and *Psychosomatic Medicine* [20]. In that study, Riehm et al. [20] found that only 25 of 76 RCTs (33%) adequately defined primary or secondary outcome analyses in the published trial reports and that only 40 trials (53%) had been registered prior to patient enrolment. Furthermore, the registrations of only 3 published trials included a defined primary outcome analysis and time point, which would allow comparison to subsequently published results.

JCCP is recognized as the preeminent APA journal for publishing important trials of psychological interventions [22]. Author instructions for other top APA journals, such as the *Journal of Abnormal Psychology*, indicate that studies focusing on treatment efficacy should be submitted to JCCP [23]. JCCP previously required compliance with CONSORT, but in 2011 replaced this policy with the requirement to adhere to the APA’s JARS guidelines. JCCP, like all other APA journals, does not require trial registration. In this context, the degree to which trials published in JCCP are registered and properly define outcome analyses in published reports is unknown. Thus, the objectives of the present study were (1) to determine the proportion of recent RCT reports published in JCCP that clearly and appropriately defined the trial’s primary and secondary outcome analyses; (2) to determine the proportion of adequately registered RCTs, according to the methods used in Riehm et al. [20] and the SPIRIT 2013 guidelines; (3) to evaluate whether published primary outcome analyses and registered primary outcomes were consistent; and (4) to compare the proportion of trials with adequately defined outcome analyses and the proportion of adequately registered RCTs to results reported by Riehm et al. [20] in top psychosomatic and behavioral medicine journals.

## Methods

### Article Selection

We used similar methods to those described by Riehm et al. [20] in their recent study of top psychosomatic and behavioral medicine journals. In the present study, we sought to identify RCTs published in JCCP between January 2013 and December 2014. To do this, citations of all articles published in JCCP in 2013–2014 were identified from the journal web page and uploaded into the citation management database RefWorks (RefWorks, RefWorks-COS, Bethesda, MD, USA), then into the systematic review program DistillerSR (Evidence Partners, Ottawa, Canada). DistillerSR was used for all coding procedures and to track results of the review process.

Based on the ICMJE definition of clinical trials, which has been used previously in studies of RCT registrations [24, 25], studies were included if they reported data from an RCT, defined as a comparative study to test the effect of an intervention on one or more health outcomes [19]. Articles that reported only secondary analyses, including subgroup analyses, were included. Studies that randomized participants into experimental conditions not intended to improve health (e.g., laughter versus mental stress conditions to assess arterial stiffness) or that primarily assessed intervention feasibility were excluded. Trials that aimed at examining the effects of a provider intervention on provider behavior only and not on the provider’s patients were also excluded. Articles that reported only mediation or moderation analyses without reporting previously unpublished primary or secondary outcome analyses, used RCT data for cross-sectional analyses only, reported on longitudinal analyses of outcomes for all participants in a trial regardless of group assignment, assessed cost-effectiveness only or analyzed only control or treatment group data were excluded.

Two investigators independently assessed article titles and abstracts for possible eligibility. Articles that were deemed potentially eligible by either reviewer underwent a full-text review by two independent investigators. Any disagreements after full-text review were resolved by consensus, involving a third investigator, as necessary.

### Data Extraction and Classification

Two investigators independently extracted and entered relevant data into a DistillerSR online database.

#### Objective 1: clearly and adequately declared outcome analyses in published articles

Published articles were classified as reporting: (1) *primary*, (2) *multiple primary (same report)*, (3) *multiple primary (different report)*, (4) *multiple primary (with statistical adjustment)*, (5) *secondary*, or (6) *undefined* outcome analyses.

An article was classified as reporting a *primary* outcome analysis if a single outcome analysis was clearly and consistently defined as primary throughout the article or, alternatively, if a single primary outcome analysis could be determined from the power analysis. Articles that measured a primary outcome variable at multiple time points in the context of a single repeated measures assessment with only one hypothesis test were classified as reporting a single *primary* outcome analysis. Studies that identified more than one primary outcome variable; that identified a single primary outcome variable, but analyzed multiple time points without specification of primacy; or that included more than two trial arms and conducted multiple between group comparisons, were classified as reporting *multiple primary (same report)* outcome analyses. If, however, studies identified more than one primary outcome analysis, but that made appropriate statistical adjustments for multiple comparisons, they were classified as reporting *multiple primary (with statistical adjustment)* outcome analyses. Studies were classified as reporting *multiple primary (different report)* outcome analyses if they identified a single primary outcome analysis, but a previous report had also declared one or more primary outcome analyses. We attempted to identify previous reports for each included RCT that were not referenced in the published RCT report by reviewing references in the included RCT and searching PubMed and PsycInfo using author names and keywords. Our methods stipulated that if a previous report that appeared to be from the same trial was identified we would determine if it referred to the same trial by comparing eligibility criteria, the intervention and comparator conditions, patient characteristics, and dates of patient enrolment of patients. Authors could be contacted if necessary. Studies classified as *primary*, *multiple primary (same report)*, *multiple primary (different report)*, and *multiple primary (with statistical adjustment)* may have also reported secondary outcome analyses, but reporting of secondary outcome analyses was not recorded when primary outcome analyses were reported.

Studies were classified as reporting *secondary* outcome analyses if the authors clearly and consistently defined one or more outcome analyses as secondary and did not report any primary outcome analyses. Studies were also classified as reporting *secondary* outcome analyses if there was a clear statement indicating that the primary or main findings of the RCT had been reported in a previous article.

Studies that did not clearly define outcome analyses as being primary or secondary were classified as reporting *undefined* analyses of outcome analyses. Studies that noted the existence of a previous report, but did not classify outcome analyses from the previous or current report as primary or secondary, were classified as reporting *undefined* outcome analyses (e.g., a report of 12-month post-intervention outcome analyses with a previous report on 6-month outcome analyses).

Studies with *primary*, *multiple primary (with statistical adjustment)*, or *secondary* outcome analyses were classified as having *adequately declared* outcome analyses, whereas studies with *multiple primary (same report)*, *multiple primary (different report)* or *undefined* outcomes were classified as having *inadequately declared* outcome analyses.

#### Objective 2: trial registration

We followed a procedure outlined by Mathieu et al. [24] and previously used by Milette et al. [25] and Riehm et al. [20]. We first attempted to retrieve trial registration data, including the registration number, from each published article. If no registration information was included in the article, we contacted the corresponding author by email to determine whether the trial had been registered and, if registered, to obtain the registry name and number of the trial. If no response was received from the corresponding author after three contact attempts, each one week apart, we searched for the studies in multiple clinical trial registries, including ClinicalTrials.gov (clinicaltrials.gov), the International Standard Randomized Trial Number (ISRCTN; www.isrctn.com), the World Health Organization (WHO) registry search portal (apps.who.int/trialsearch), and the registry from the country of the first author (e.g., Netherlands Trial Register [www.trialregister.nl]). To identify registry records, we performed a search using key terms from the published article, then attempted to match the principal investigator, funding source, intervention, control group, and design from the article to the registrations obtained in the search. If this method did not uncover a registration number, the published article was coded as not registered. The search for trial registrations was performed on January 16, 2015.

For both registered and unregistered trials, we attempted to extract trial start and end dates and participant enrollment dates from the publication to determine if the trial should have been registered per ICMJE policy. If the publication did not provide these dates, for registered trials, we extracted them from the registration record. For trials in the ISRCTN registry, the “date applied” was used at the date of registration and was extracted from the registration. For studies in the ClinicalTrials.gov registry, the “first received” date was extracted from the registration. From the information provided in the registration, we determined the proportion of published RCTs that were registered versus not registered. We also assessed the proportion that began enrolling patients July 1, 2005 or later and should have been registered prior to patient enrollment based on ICMJE guidelines.

We assessed the adequacy of primary outcome declaration in trial registrations for all published RCTs, except those that only reported secondary outcome analyses, using two methods. First, using a method described by Mathieu et al. [24] and used by Milette et al. [25] and Riehm et al. [20], registered studies were classified as having *adequately* or *inadequately registered* outcomes. Per ICMJE policy, trials that were ongoing as of July 1^st^, 2005 had to be registered prior to September 13^th^, 2005 and prior to trial completion, but not prior to enrolling trial participants. Studies that started after July 1^st^, 2005 must have been registered before participant enrollment began.

In our assessment of registration adequacy, we only included studies that began enrolling participants after July 1, 2005. To be classified as an *adequately registered* trial, studies had to (1) have been registered prior to beginning participant enrolment; and (2) have specified one primary outcome variable in the registry with a clear description and time frame of assessment, or multiple primary outcomes with a plan for statistical adjustment. If these criteria were not met, the registered RCT was classified as having *inadequately registered* outcomes.

Second, we evaluated the completeness of registered primary outcomes according to SPIRIT 2013 guidelines [21]. For each registered outcome, we evaluated whether the following elements were provided; (1) the specific measurement variable (e.g., Beck Depression Inventory score, not simply “depression”); (2) the participant-level analysis metric (e.g., change from baseline, final value, time to event); (3) the method of aggregation (e.g., mean, proportion above a cutoff); and (4) the specific measurement time point of interest for analysis.

For both the method used in other recent studies (e.g. [20]) and the SPIRIT method of assessing adequacy of outcome declaration, trial registrations that specified more than one primary outcome were considered to have adequately registered the outcome only if a plan for statistical adjustment for multiple comparisons was included in the registration or if the same set of primary outcomes that were registered were analyzed with proper statistical adjustment in the published report. Trial registrations that did not specify a single primary time point for analysis were considered to have met the time point criterion only in the context of a planned analysis that examined change across all time points with a single analysis that tested a single hypothesis. If there were changes in the study registration records of a trial, we extracted data from the last registration update prior to the beginning of participant enrolment.

#### Objective 3: comparison of registered primary outcomes to published primary outcome analyses

For each trial that was *adequately registered* and included at least one primary outcome analysis in the published report, we compared registered primary outcomes to published primary outcome analyses. First, studies *adequately registered* as per criteria used in recent studies (e.g. [20]) were classified as reporting *consistent* outcome analyses if the published primary outcome analyses were consistent with the primary outcomes specified in the trial registration. Studies were classified as reporting *discrepant* outcome analyses if the published primary outcome analyses were not consistent with the primary outcome analyses specified in the trial registration.

#### Objective 4: comparison of results to Riehm and al. [20]

We compared the proportion of studies with *adequately declared* published outcome analyses and the proportion of studies that were *adequately registered* as per methods used in recent studies (e.g. [20]) to the results obtained by Riehm and al. [20] from top psychosomatic and behavioral medicine journals. Proportions of adequate published outcome analysis definitions and trial registrations were compared using a chi-square test with alpha = 0.05. We used a Fisher’s exact test to compare the proportion of adequately registered trials since there were expected counts per cell of < 5 in some cases.

## Results

### Article Selection

**Fig 1:**
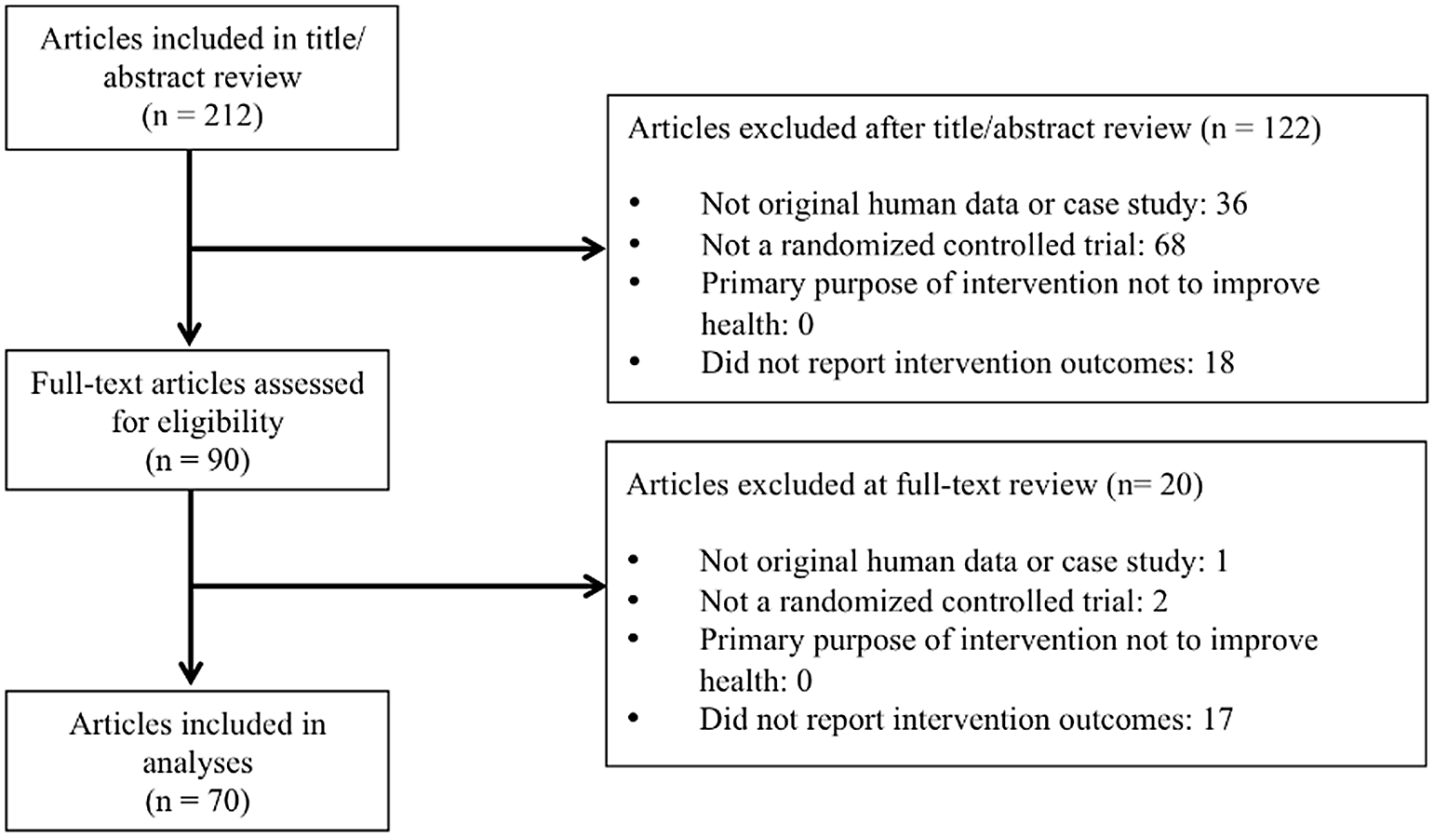
Article Selection.

A total of 212 articles were published in JCCP in 2013–2014. Of these, 122 were excluded after title-abstract review, and 20 more were excluded after full-text review. Thus, 70 RCTs were included (Fig 1), 31 from 2013 and 39 from 2014. The outcome declaration and registration classifications for all included articles are found in S2 Table.

#### Objective 1: clearly and adequately declared outcome analyses in published articles

Only 12 of the 70 RCTs (17.1%) were classified as having *adequately declared* outcome analyses in the published article, including 9 (12.9%) with *adequately declared primary* outcome analyses, one (1.4%) with *adequately declared multiple primary (with statistical adjustment)* outcome analyses, and 2 (2.9%) with *adequately declared secondary* outcome analyses. Of the 58 articles (82.9%) that had *inadequately declared* outcome analyses, 44 (62.9%) declared *multiple primary* outcomes without appropriate statistical adjustment and 14 (20.0%) had *undefined* outcome analyses (Table 1).

**Table 1:** Outcome Analysis Declaration in Published Reports of Randomized Controlled Trials in JCCP.

	2013: N (% of total)	2014: N (% of total)	Both Years: N (% of total)
**Adequately declared outcome analyses:**	**5 (16.1%)**	**7 (17.9%)**	**12 (17.1%)**
Primary	5	4	9 (12.9%)
Multiple primary (with statisticaladjustment)	0	1	1 (1.4%)
Secondary	0	2	2 (2.9%)
**Inadequately declared outcome analyses:**	**26 (83.9%)**	**32 (82.1%)**	**58 (82.9%)**
Multiple primary (same report)	23	21	44 (62.9%)
Multiple primary (different report)	0	0	0 (0.0%)
Undefined	3	11	14 (20.0%)
**Total**	**31 (100%)**	**39 (100%)**	**70 (100%)**

#### Objective 2: trial registration

Registration status of included RCTs is shown in Table 2. Of the 70 articles reviewed, 39 (55.7%) reported on registered RCTs and 31 (44.3%) on unregistered RCTs. Of the 39 articles that reported on a registered RCT, 14 (35.9%) provided registration information in the publication, whereas registration information was obtained from authors or by searching trial registries for 25 (64.1%).

**Table 2:** Registration of Published RCTs and Pre-Enrolment Registration Requirement per ICMJE Requirements.

	2013	2014	Both Years (N, % of total)
**Unregistered RCT Publications**	**15 (48.4%)**	**16 (41.0%)**	**31 (44.3%)**
**Registered RCT Publications**	**16 (51.6%)**	**23 (59.0%)**	**39 (55.7%)**
Pre-enrolment registration required	15	22	37 (52.9%)
Pre-enrolment registration not required	1	1	2 (2.9%)
Could not assess registration requirement	0	0	0 (0%)
**Total**	**31 (100%)**	**39 (100%)**	**70 (100%)**

*Note*. ICMJE = International Committee of Medical Journal Editors

As shown in Table 2, among the 39 registered trials, 37 were required to be registered pre-enrolment and were considered in our assessment of registration adequacy, whereas 2 began prior to July 2005 and were not considered. Of the 37 registered trials reviewed for adequacy of outcome registration, one article reported on only *adequately declared secondary* outcome analyses in the JCCP publication. Of the other 36 that published primary outcome analyses in the JCCP article, there were 9 that registered pre-enrolment (25.0%), but only 2 of the 36 (5.6%) were registered pre-enrolment, clearly defined a primary outcome variable and time point, and, thus, were classified as registering *adequately registered* outcomes per methods used by recent studies, including Riehm et al. [20]. None of the 29 trials that registered after enrollment began included an adequately declared primary outcome variable and time point in the registration.

Based on the SPIRIT guidelines evaluation of trial registrations [21], there were 3 trials (8.3%) that registered pre-enrolment and adequately specified a primary outcome variable; 2 (5.6%) with pre-enrolment registration, a primary outcome variable, and time point of interest for analysis; one (2.8%) with pre-enrolment registration, a primary outcome variable, time point, and analysis metric; but none with all of these plus a method of aggregation.

#### Objective 3—comparison of registered primary outcomes to published primary outcome analyses

Of the 2 articles [26–27] that reported on RCTs with *adequately registered* outcomes per Riehm et al. [20] criteria, one article published outcome analyses in JCCP in a manner that was *consistent* with registered outcomes. In the other article, reported outcome analyses and registered outcomes were *discrepant* (See S2 Table).

#### Objective 4—comparison of JCCP results to results from trials published in top psychosomatic and behavioral medicine journals (Riehm et al. [20])

There was a significantly lower proportion of published RCTs with *adequately declared* outcome analyses in JCCP (12 of 70; 17.1%) compared to RCTs during the same time period published in four top psychosomatic and behavioral medicine journals (25 of 76, 32.9%; **χ** ^2^(1) = 4.78, *p* = 0.029). A slightly higher proportion of JCCP trials were registered (39 of 70; 55.7%) compared to journals from the psychosomatic and behavioral medicine journals (40 of 76; 52.6%), although the difference was not statistically significant (**χ** ^2^(1) = 0.14, *p* = 0.709). Only 2 of 70 JCCP trials (2.9%) were *adequately registered* compared to 3 of 76 (3.9%) in psychosomatic and behavioral medicine journals (*p* > 0.99).

As shown in Table 3, we conducted a sensitivity analysis that compared RCTs published in JCCP and those published in the three non-APA journals reviewed by Riehm et al. [20], *Annals of Behavioral Medicine*, *Journal of Psychosomatic Research* and *Psychosomatic Medicine*, excluding *Health Psychology*, which is an APA journal. Results for the proportion of adequately declared published outcome analyses (JCCP = 17.1%, non-APA = 27.9%; *p* = 0.17) and proportion of registered trials (JCCP = 55.7%, non-APA = 67.4%; *p* = 0.22) were not substantively different from the main analyses.

**Table 3:** Comparing Outcome Declaration and Registration of Published RCTs in JCCP and Non-APA Psychosomatic and Behavioral Medicine Journals.

	JCCP: n/N (%)	Non-APA Journals: n/N (%)	P-value
**Adequately declared outcomes**	12/70 (17.1%)	12/43 (27.9%)	0.17
**Registered RCT Publications**	39/70 (55.7%)	29/43 (67.4%)	0.22

## Discussion

Of 70 RCT publications in JCCP from 2013 and 2014 that were reviewed, only 17% adequately defined primary or secondary outcome analyses in the published reports. Approximately half of the trials were registered, but less than 15% were registered prior to enrolling patients, and the quality of outcome definitions in the registry entries was generally poor. Only 2 of 70 trials adequately registered a single primary outcome variable and time point of assessment prior to enrolling patients. However, in one of the two trials, the published outcome analysis was not consistent with the registered outcome.

The proportion of adequately defined outcome analyses for trials published in JCCP has increased since a previous review reported that no RCTs published between 1992 and 2002 in JCCP adequately specified primary or secondary outcome analyses [28]. However, JCCP appears to lag behind some other similar journals in this regard currently, even though JCCP is generally recognized as APA’s primary outlet to publish trials and even though JCCP’s impact factor (2014 impact factor = 5.3) is higher than the other journals that performed somewhat better. The methods used in the current study were the same as the methods from a review by Riehm et al. [20] that examined similar outcomes from RCTs published in four top psychosomatic and behavioral medicine journals in 2013–2014, namely *Annals of Behavioral Medicine* (2014 impact factor = 4.1), *Health Psychology* (2014 impact factor = 3.6), *Journal of Psychosomatic Research* (2014 impact factor = 2.7) and *Psychosomatic Medicine* (2014 impact factor = 3.5), in 2013–2014. Riehm et al. [20] found that 33% of the 76 trials reviewed had adequately declared primary or secondary outcome analyses, which is approximately double the rate for JCCP (17%). The 55% of trials published in JCCP that were registered prior to enrolling participants was similar to results in Riehm et al [20], although the quality of outcome definitions in trial registrations was poor in both studies, with less than 5% of trials classified as having *adequately registered* outcomes based on the proper designation of primary outcome variables and assessment time points prior to patient enrollment. No RCT reviewed in either study fully complied with SPIRIT criteria [21] for trial registrations, which additionally requires specification of the analysis metric (e.g., change from baseline, final value) and the method of aggregation (e.g., mean, proportion above or below a cutoff threshold).

Differences in registration rates and adequate outcome analysis definitions in publications across journals may be related to differences in author guidelines. Of the four psychosomatic and behavioral medicine journals and JCCP, *Annals of Behavioral Medicine*, *Journal of Psychosomatic Research*, and *Psychosomatic Medicine* require trial registration, and the percentage of trial registration in those journals ranged from 60% to 78%. The percentage was lower in the two APA journals, *Health Psychology* (33%) and JCCP (55%), which do not require trial registration. Regarding outcome definitions in published studies, all four psychosomatic and behavioral medicine journals require compliance with CONSORT [29–32], and the percentage of trials in those journals with adequately declared outcome analyses ranged from 27% to 39%. Those percentages are substantially higher than the 17% in JCCP, which requires the use of the JARS guideline [22], but not CONSORT. These results are consistent with prior evidence that suggests that implementation of rules and requirements by journals tends to have a modest effect on trial reporting practices. A 2012 Cochrane review, for instance, reported that journal endorsement of CONSORT was associated with improved reporting of published RCT results, but that the effect was small [33].

The shortcomings identified in the present study with respect to trial registration and outcome reporting likely reflect some combination of low quality trial design and low quality reporting. Low quality trial design, including the failure to pre-specify a primary analysis, can increase the likelihood that estimates of intervention effectiveness are biased due to selective reporting. If it is not clear whether or not authors pre-specified a primary outcome due to low quality reporting, on the other hand, it is difficult to evaluate the results of a trial properly, but not necessarily indicative of bias.

Efforts to improve the transparency and completeness of the reporting of trial results have focused most on regulated interventions, such as drugs, biologics, and medical devices. Regulatory agencies, including the United States Food and Drug Administration and the European Medicines Agency, have implemented legal mandates for trial registration and reporting of results [34]. Non-regulated health care interventions, including psychological interventions, have received less attention, but this is changing and calls have been made for increased regulatory attention as well as for external incentives to increase compliance with registration and reporting mandates [34]. In addition to encouraging journal editors to implement registration and reporting requirements, potential external incentives might include requirements for trial registration prior to granting of ethics approval, which has been done in the UK since 2013, regulations from funding agencies that link release of funds to registration, and adaptation of polices by professional associations [34].

Many researchers may not be aware of the need to register trials and clearly outline a trial design that includes appropriately defined trial outcome analyses prior to enrolling patients. For instance, one study which interviewed 59 investigators of trials included in Cochrane systematic reviews found that the majority of investigators who excluded non-significant results from published trial reports were largely unaware of the effect that selectively reporting positive results from individual studies can have on the overall evidence base [35]. Indeed, intervention research is often couched in terms of a search for positive evidence to demonstrate the effectiveness of novel interventions, instead of prioritizing high-quality evaluations of health care interventions, regardless of whether results suggest effectiveness or not [36, 37]. Consistent with this, the general public and popular press overwhelmingly emphasize positive health care trials that are suggestive of new treatment opportunities, with less coverage given to negative trials [38]. Similarly, journal editors and reviewers favor trials with statistically significant results compared to trials with non-significant results [39]. Some researchers have reported that they feel that it is not worth the time to try to publish negative results, that negative results are not important, and that reports of negative results will be rejected by journals [40]. Other factors that may lead to the non-reporting of negative results include allegiances to particular treatment modalities [41] or the perception that generating positive results, which are more likely to be published in high-impact journals [39], may be linked to academic promotion and career success, for instance.

Ideally, both educational approaches and external incentives can be used to improve the quality of trial registration and outcome definitions and reporting of psychological interventions, and no single journal can perform these tasks alone. Nonetheless, JCCP, the premier trials journal among APA journals with a long history of promoting and educating the field about the best research practices [42], is well positioned to take a leadership role in addressing these issues. JCCP should adapt a policy that requires pre-patient enrolment registration of trials considered for publication in the journal and should provide reviewers with tools to incorporate enforcement of the policy as part of the peer review process. Similarly, it is important that authors are aware of expectations regarding definitions and reporting of primary and secondary outcome analyses and that reviewers and editors carefully evaluate the adequacy of outcome analysis definitions and whether outcome analyses are consistent with pre-trial registration. Although JARS does not provide guidance on the importance of carefully selecting a single primary outcome analysis and to avoid Type I error inflation from multiple analyses, CONSORT does and should be included as part of JCCP author guidelines. *Health Psychology*, another APA journal, has already implemented CONSORT. In order to help reviewers incorporate an assessment of outcome analysis definitions, a peer review package could include questions pertaining to the specificity of outcome analysis reporting. For example, was a single primary outcome analysis specified? If not, were statistical adjustments made for multiple outcome analyses? In addition, questions could also be asked regarding the consistency of reporting. Was the same primary outcome analysis defined consistently across the various documents? If the primary outcome analysis was not consistent with registered outcomes, did the authors provide justification for the change, and, if so, is this justification acceptable [20]?

Besides journals, academic and funding institutions must get more involved in incentivizing trial registration and transparency in outcome reporting [9]. For example, funding agencies can require prospective trial registration and access to trial results and could ultimately suspend funding until certain conditions are met [34]. Hence, journal editors, publishers, funding agencies and professional and academic associations can all play a role in advocating for the implementation of trial registration.

The results of the present study should be interpreted in light of relevant limitations. First, due to the relatively short time span of that the study covered, only 70 RCTs were reviewed. Secondly, although our findings were informative in terms of outcome analysis definition and trial registration rates in JCCP, our results may not generalize to other APA journals that also publish trials.

In summary, most trials published in JCCP from 2013–2014 did not adequately define primary or secondary outcome analyses in the published trial reports, and nearly half of the trials were not registered with less than 15% registered prior to enrolling patients. There was only one study that adequately registered trial outcomes prior to enrolling patients and published outcome analyses that were consistent with registered outcomes. JCCP is well established as a standard bearer for promoting high-quality research on psychological interventions and is well positioned to positively influence the field. This should be done by endorsing trial registration and CONSORT requirements and by providing peer reviewers with the tools to ensure that JCCP effectively implements these requirements. Beyond JCCP, the scientific community, including researchers, other journals, academic institutions, and funders would serve the public better if more attention were paid to the accurate and transparent reporting of clinical trials of psychological treatments.

## Supporting Information

S1 Table Examples of academic medical center websites that describe online trial registration. (DOCX)

S2 Table Characteristics of included RCTs. (DOCX)

## References

[1] APA. Evidence-based practice in psychology. American Psychologist. 2006;61(4):271–85. 16719673 10.1037/0003-066X.61.4.271

[2] Altman DG, Schulz KF, Moher D, Egger M, Davidoff F, Elbourne D, et al The revised CONSORT statement for reporting randomized trials: explanation and elaboration. Annals of Internal Medicine. 2001;134(8):663–94. 11304107 10.7326/0003-4819-134-8-200104170-00012

[3] Murray GD. Promoting good research practice. Statistical methods in medical research. 2000;9(1):17–24. 10826155 10.1177/096228020000900103

[4] Schulz KF, Grimes DA. Multiplicity in randomised trials I: endpoints and treatments. Lancet. 2005;365(9470):1591–5. 15866314 10.1016/S0140-6736(05)66461-6

[5] Friedman LM, Furberg C, DeMets DL. Fundamentals of clinical trials: Springer; 2010.

[6] Schulz., Altman DG, Moher D. CONSORT 2010 statement: updated guidelines for reporting parallel group randomized trials. Annals of Internal Medicine. 2010;152(11):726–32. 10.7326/0003-4819-152-11-201006010-00232 20335313

[7] Pocock SJ. Clinical trials with multiple outcomes: a statistical perspective on their design, analysis, and interpretation. Controlled clinical trials. 1997;18(6):530–45; discussion 46–9. 9408716 10.1016/s0197-2456(97)00008-1

[8] Schulz KF, Grimes DA. Sample size calculations in randomised trials: mandatory and mystical. Lancet. 2005;365(9467):1348–53. 15823387 10.1016/S0140-6736(05)61034-3

[9] Chan AW, Hrobjartsson A, Haahr MT, Gotzsche PC, Altman DG. Empirical evidence for selective reporting of outcomes in randomized trials: comparison of protocols to published articles. JAMA. 2004;291(20):2457–65. 15161896 10.1001/jama.291.20.2457

[10] Jadad AR, Enkin M.W. Bias in Randomized Controlled Trials Randomized Controlled Trials: Questions, Answers, and Musings. second edition ed: Blackwell Publishing; 2007.

[11] Page MJ, McKenzie JE, Kirkham J, Dwan K, Kramer S, Green S, et al Bias due to selective inclusion and reporting of outcomes and analyses in systematic reviews of randomised trials of healthcare interventions. The Cochrane database of systematic reviews. 2014;10:Mr000035 10.1002/14651858.MR000035.pub2 25271098 PMC8191366

[12] Sterne JAC, Egger M, Moher D (editors). Chapter 10: Addressing reporting biases In: Higgins JPT, Green S (editors). *Cochrane Handbook for Systematic Reviews of Intervention*. Version 5.1.0 (updated March 2011). The Cochrane Collaboration, 2011 Available from www.cochrane-handbook.org.

[13] Page MJ, McKenzie JE, Forbes A. Many scenarios exist for selective inclusion and reporting of results in randomized trials and systematic reviews. Journal of Clinical Epidemiology. 2013;66(5):524–37. 10.1016/j.jclinepi.2012.10.010 23337785

[14] Saquib N, Saquib J, Ioannidis JP. Practices and impact of primary outcome adjustment in randomized controlled trials: meta-epidemiologic study. BMJ. 2013;347:f4313 10.1136/bmj.f4313 23851720 PMC3709831

[15] Moher D, Hopewell S, Schulz KF, Montori V, Gotzsche PC, Devereaux PJ, et al CONSORT 2010 explanation and elaboration: updated guidelines for reporting parallel group randomised trials. International Journal of Surgery. 2012;10(1):28–55. 10.1016/j.ijsu.2011.10.001 22036893

[16] Boutron I, Moher D, Altman DG, Schulz KF, Ravaud P. Extending the CONSORT statement to randomized trials of nonpharmacologic treatment: explanation and elaboration. Annals of Internal Medicine. 2008a;148(4):295–309. 18283207 10.7326/0003-4819-148-4-200802190-00008

[17] Boutron I, Moher D, Altman DG, Schulz KF, Ravaud P. Methods and Processes of the CONSORT Group: Example of an Extension for Trials Assessing Nonpharmacologic Treatments. Annals of Internal Medicine. 2008b;148(4):W-60. 18283201 10.7326/0003-4819-148-4-200802190-00008-w1

[18] APA. Reporting Standards for Research in Psychology. Why Do We Need Them? What Might They Be? American Psychologist. 2008;63(9):839–51. 10.1037/0003-066X.63.9.839 19086746 PMC2957094

[19] De Angelis C, Drazen JM, Frizelle FA, Haug C, Hoey J, Horton R, et al Clinical trial registration: a statement from the International Committee of Medical Journal Editors. The New England journal of medicine. 2004;351(12):1250–1. 15356289 10.1056/NEJMe048225

[20] Riehm KE, Azar M, Thombs BD. Transparency of outcome reporting and trial registration of randomized controlled trials in top psychosomatic and behavioral health journals: A 5-year follow-up. Journal of Psychosomatic Research. 2015. 10.1016/j.jpsychores.2015.04.010 25956011

[21] Chan AW, Tetzlaff JM, Götzsche PC, Altman DG, Mann H, Berlin JA, et al SPIRIT 2013 explanation and elaboration: guidance for protocols of clinical trials. BMJ. 2013;346:e7586 10.1136/bmj.e7586 23303884 PMC3541470

[22] APA. Journal of Consulting and Clinical Psychology: Instructions to Authors: American Psychological Association; 2015 [cited 2015 July]. Available from: http://www.apa.org/pubs/journals/ccp/?tab=4.

[23] APA. Journal Of Abnormal Psychology: Instructions to Authors Washington DC: American Psychological Association; 2015 [cited 2015 July]. Available from: http://www.apa.org/pubs/journals/abn/index.aspx?tab=4.

[24] Mathieu S, Boutron I, Moher D, Altman DG, Ravaud P. Comparison of registered and published primary outcomes in randomized controlled trials. JAMA. 2009;302(9):977–84. 10.1001/jama.2009.1242 19724045

[25] Milette K, Roseman M, Thombs BD. Transparency of outcome reporting and trial registration of randomized controlled trials in top psychosomatic and behavioral health journals: A systematic review. Journal of Psychosomatic Research. 2011;70(3):205–17. 10.1016/j.jpsychores.2010.09.015 21334491

[26] Olthuis JV, Watt MC, Mackinnon SP, Stewart SH. Telephone-delivered cognitive behavioral therapy for high anxiety sensitivity: a randomized controlled trial. Journal of Consulting and Clinical Psychology. 2014;82(6):1005–22. 10.1037/a0037027 24911423

[27] Rogers K, Banis M, Falkenstein MJ, Malloy EJ, McDonough L, Nelson SO, et al Stepped care in the treatment of trichotillomania. Journal of Consulting and Clinical Psychology. 2014;82(2):361–7. 10.1037/a0035744 24491078 PMC3966933

[28] Cook JM, Hoffmann K, Coyne JC, Palmer SC. Reporting of Randomized Clinical Trials in the Journal of Consulting and Clinical Psychology 1992 and 2002: Before Consort and Beyond. Scientific Review of Mental Health Practice. 2007;5(1).

[29] Kaplan RM. Changes in Annals’ editorial policies. Annals of Behavioral Medicine. 2002;24(3):167–8.

[30] Arthur A. Editorial: Modification to “Instructions to authors”. 2003.

[31] Sheps DS. Changes in the Air atPsychosomatic Medicine. Psychosomatic Medicine. 2003;65(4):499–500.

[32] Creed F, Levenson JL. Raising standards of research reporting in the Journal. Journal of Psychosomatic Research. 2011;70(3):203–4. 10.1016/j.jpsychores.2011.01.011 21334490

[33] Turner L, Shamseer L, Altman DG, Schulz KF, Moher D. Does use of the CONSORT Statement impact the completeness of reporting of randomised controlled trials published in medical journals? A Cochrane review. Systematic reviews. 2012;1:60 10.1186/2046-4053-1-60 23194585 PMC3564748

[34] Dal-Ré R, Bracken MB, Ioannidis JP. Call to improve transparency of trials of non-regulated interventions. BMJ. 2015;350:h1323 10.1136/bmj.h1323 25820265

[35] Smyth R, Kirkham J, Jacoby A, Altman D, Gamble C, Williamson P. Frequency and reasons for outcome reporting bias in clinical trials: interviews with trialists. BMJ. 2011;342:c7153 10.1136/bmj.c7153 21212122 PMC3016816

[36] Brice A, Chalmers I. Medical journal editors and publication bias. BMJ. 2013;347:f6170 10.1136/bmj.f6170 24150668

[37] Chalmers I. Proposal to outlaw the term “negative trial”. British Medical Journal (Clinical research ed). 1985;290(6473):1002-.3919855

[38] Koren G, Klein N. Bias against negative studies in newspaper reports of medical research. JAMA. 1991;266(13):1824–6. 1890712

[39] Wager E, Williams P. "Hardly worth the effort"? Medical journals' policies and their editors' and publishers' views on trial registration and publication bias: quantitative and qualitative study. BMJ. 2013;347:f5248 10.1136/bmj.f5248 24014339 PMC3805489

[40] Song F, Loke Y, Hooper L. Why are medical and health-related studies not being published? A systematic review of reasons given by investigators. PLoS One. 2014;9(10):e110418 10.1371/journal.pone.0110418 25335091 PMC4198242

[41] Luborsky L, Diguer L, Seligman DA, Rosenthal R, Krause ED, Johnson S, et al The Researcher's Own Therapy Allegiances: A “Wild Card” in Comparisons of Treatment Efficacy. Clinical Psychology: Science and Practice. 1999;6(1):95–106.

[42] Nezu AM. Editorial. Journal of Consulting and Clinical Psychology. 2011;79(1):1–5. 10.1037/a0022353 21261429

