# Supplementary material for: Transparency of Outcome Reporting and Trial Registration of Randomized Controlled Trials Published in the Journal of Consulting and Clinical Psychology
Source: PLoS One. 2015 Nov 18;10(11):e0142894. doi: 10.1371/journal.pone.0142894 (PMC4651548; doi:10.1371/journal.pone.0142894)
Supplement: S1 Table — (DOCX) [file pone.0142894.s001.docx]

**S1 Table. Examples of academic medical center websites that describe online trial registration.**

| **University of Michigan** | www.med.umich.edu/medschool-regulatory/Policies/Clinicaltrials-gov.html |
| --- | --- |
| **University of California, Irvine** | www.research.uci.edu/compliance/human-research-protections/researchers/guidelines-for-registering-in-a-clinicaltrialsgov-registry.html |
| **Duke University** | www.medschool.duke.edu/research/clinical-and-translational-research/duke-office-clinical-research/irb-and-institutional-approval/registering-trial-clinicaltrialsgov-investigator-initiated-research |
| **University of Vermont** | www.uvm.edu/irb/ClinicalTrialRegistryInstruction.pdf |
| **Georgia Regents University** | www.gru.edu/research/irboffice/irb/clinical_trials_registration_at_clinical_trials_gov.pdf |
