# Supplementary material for: Transparency of Outcome Reporting and Trial Registration of Randomized Controlled Trials Published in the Journal of Consulting and Clinical Psychology
Source: PLoS One. 2015 Nov 18;10(11):e0142894. doi: 10.1371/journal.pone.0142894 (PMC4651548; doi:10.1371/journal.pone.0142894)
Supplement: S2 Table — (DOCX) [file pone.0142894.s002.docx]

**S2 Table. Characteristics of included RCTs**

|  |  | **Published Outcome Analyses Per Milette et al. (2011) and Riehm et al. (2015) Methods** | |  | **Trial Registration** | | |
| --- | --- | --- | --- | --- | --- | --- | --- |
| **First Author, Year** | **Purpose** | **Reported Outcome Analyses** | **Adequate / Inadequate Outcome Analysis Reporting** |  | **Registration Status** | **Registry Name and Number** | **Adequate / Inadequate Outcome Registration and Consistency of Registered/Published Results** |
| Abikoff, 2013 | To compare the efficacy of 2 behavioral interventions to wait-list control to ameliorate organization, time management, and planning difficulties in 3rd- to 5th-grade children with attention-deficit/hyperactivity disorder. | Multiple primary (same report) | Inadequate |  | Registered^†^ | NCT00381407 | Inadequate |
| Anderson, 2013 | To compare virtual reality exposure therapy to in vivo exposure for social anxiety disorder. | Multiple primary (same report)^‡^ | Inadequate |  | Not registered | Not applicable | Not applicable |
| Armitage, 2014 | To test the ability of a very brief intervention based on self-affirmation theory to reduce alcohol consumption in a sample of adolescents. | Primary | Adequate |  | Not registered | Not applicable | Not applicable |
| Bodenmann, 2014 | To test an instructional DVD versus a DVD with telephone coaching compared to a wait-list control on couples' communication and coping. | Undefined | Inadequate |  | Not registered | Not applicable | Not applicable |
| Bombardier, 2013 | To compare a 12-week physical activity counseling intervention delivered by phone to a wait-list control group. | Primary | Adequate |  | Not registered | Not applicable | Not applicable |
| Brief, 2013 | To evaluate the efficacy of a self-management web intervention (VetChange) based on motivational and cognitive-behavioral principles to reduce alcohol consumption, alcohol-related problems, and post-traumatic stress disorder symptoms in returning combat veterans. | Multiple primary (same report) | Inadequate |  | Not registered | Not applicable | Not applicable |
| Chorpita, 2013 | To test 3 treatment conditions, (1) standard use of 1 or more of 3 manualized treatments; (2) modular, which involved more flexibility; and (3) usual care on mental health symptoms. | Multiple primary (same report) | Inadequate |  | Registered^†^ | NCT01178554 | Inadequate |
| Christensen, 2014 | To examine the benefit of adding an internet-delivered behavior therapy to a buprenorphine medication program and voucher-based motivational incentives. | Multiple primary (same report) | Inadequate |  | Registered^*^ | NCT00929253 | Inadequate |
| Chronis-Tuscano, 2013 | To examine the preliminary efficacy of an integrated treatment targeting parenting and depressive symptoms for mothers of children with attention deficit hyperactivity disorder. | Undefined | Inadequate |  | Registered^†^ | NCT00316290 | Inadequate |
| Cordova, 2014 | To assess the efficacy of the Marriage Checkup for improving relationship health and intimacy. | Multiple primary (same report) | Inadequate |  | Not registered | Not applicable | Not applicable |
| Craske, 2014 | To test in a 3-arm trial cognitive-behavioral therapy and acceptance and commitment therapy versus a waiting list control group for social phobia. | Multiple primary (same report) | Inadequate |  | Registered^†^ | NCT00872820 | Inadequate |
| Donohue, 2014 | To test the effect of family behavior therapy to reduce child maltreatment potential among mothers referred to child protective services for drug abuse or dependence and child neglect. | Multiple primary (same report) | Inadequate |  | Not registered | Not applicable | Not applicable |
| Garland, 2014 | To conduct an early-stage randomized controlled trial of Mindfulness-Oriented Recovery Enhancement, a multimodal intervention designed to simultaneously target mechanisms underpinning chronic pain and opioid misuse. | Multiple primary (same report) | Inadequate |  | Registered^†^ | NCT01505101 | Inadequate |
| Godley, 2014 | To test whether contingency management, assertive continuing care, and their combination would each be more effective than usual continuing care in adolescents with alcohol and other drug use disorders. | Multiple primary (same report) | Inadequate |  | Registered^†^ | NCT01085188 | Inadequate |
| Granholm, 2014 | To compare cognitive behavioral social skills training to an active goal-focused supportive contact control for improving function and reducing negative symptoms in patients with schizophrenia or schizoaffective disorder. | Primary | Adequate |  | Registered^*^ | NCT00338975 | Inadequate |
| Harvey, 2014 | To compare behavior therapy, cognitive therapy and full cognitive behavior therapy for persistent insomnia. | Multiple primary (same report) | Inadequate |  | Registered^†^ | NCT00869934 | Inadequate |
| Hayes-Skelton, 2013 | To examine whether a treatment combining mindfulness- and acceptance-based strategies with behavioral approaches would improve outcomes in generalized anxiety disorder compared to an empirically supported treatment. | Multiple primary (same report) | Inadequate |  | Registered^†^ | NCT00073632 | Inadequate |
| Hektner, 2014 | To examine the long-term effects of the Early Risers "Skills for Success" Conduct Problems Prevention Program to promote adaptive psychological development in kindergarten children. | Multiple primary (same report) | Inadequate |  | Not registered | Not applicable | Not applicable |
| Ironson, 2013 | To examine the efficacy of a brief written trauma disclosure intervention on posttraumatic stress, depression, HIV-related physical symptoms, and biological markers of HIV disease progression. | Multiple primary (same report) | Inadequate |  | Registered^†^ | NCT00067704 | Inadequate |
| Kerr, 2014 | To test the effects of multidimensional foster care on long-term trajectories of suicidal ideation and depressive symptoms. | Multiple primary (same report) | Inadequate |  | Registered^†^ | NCT01341626 | Inadequate |
| Kiernan, 2013 | To examine whether learning a novel set of "stability skills" before losing weight improved long-term weight management. | Primary | Adequate |  | Registered^†^ | NCT00626457 | Inadequate |
| Kushner, 2013 | To compare cognitive-behavioral therapy to progressive muscle relaxation training for symptoms of anxiety and alcohol use. | Multiple primary (same report) | Inadequate |  | Not registered | Not applicable | Not applicable |
| LaBrie, 2013 | To compare the efficacy of a web-based personalized normative feedback intervention to the web-based Brief Alcohol Screening and Intervention for College Students intervention and a repeated-assessment control for reducing risky drinking and associated consequences. | Multiple primary (same report) | Inadequate |  | Not registered | Not applicable | Not applicable |
| Lee, 2013 | To evaluate the efficacy of an in-person brief motivational enhancement intervention for reducing marijuana use and related consequences among frequently using college students. | Multiple primary (same report) | Inadequate |  | Not registered | Not applicable | Not applicable |
| Lee, 2014 | To evaluate the efficacy of event-specific prevention strategies for reducing spring break drinking among college students, compared to general prevention strategies and an assessment-only control group, as well to evaluate inclusion of peers in interventions and mode of intervention delivery (in-person versus web). | Undefined | Inadequate |  | Not registered | Not applicable | Not applicable |
| Lewis, 2014 | To evaluate the efficacy of personalized normative feedback on college student alcohol-related risky sexual behavior. | Undefined | Inadequate |  | Not registered | Not applicable | Not applicable |
| Liber, 2013 | To investigate the effectiveness of a school-based targeted intervention program for disruptive behavior. | Multiple primary (same report) | Inadequate |  | Registered^*^ | NTR1352 | Inadequate |
| Lumley, 2014 | To test the independent and combined effects of coping skills training and written emotional disclosure in rheumatoid arthritis. | Multiple primary (same report) | Inadequate |  | Registered^*^ | NCT00088764 | Inadequate |
| Martens, 2013 | To test the efficacy of 2 single-component, in-person, brief alcohol interventions, personalized normative feedback and protective behavioral strategies feedback in college students. | Multiple primary (same report) | Inadequate |  | Registered^*^ | NCT01168726 | Inadequate |
| McDevitt-Murphy, 2014 | To test the efficacy of 2 brief interventions for alcohol misuse in a sample of combat veterans. | Undefined | Inadequate |  | Not registered | Not applicable | Not applicable |
| McKay, 2013 | To compare treatment as usual, treatment as usual and telephone monitoring and counseling and treatment as usual and telephone monitoring and counseling plus incentives in cocaine dependent patients. | Multiple primary (same report) | Inadequate |  | Registered^†^ | NCT00685659 | Inadequate |
| Mikami, 2013 | To compare contingency management training to a novel treatment that included peer training for behavioral management of children with attention deficit hyperactivity disorder. | Multiple primary (same report) | Inadequate |  | Registered^†^ | NCT01133028 | Inadequate |
| Monti, 2014 | To investigate the relative efficacy of a significant other-enhanced motivational intervention compared with an individual motivational intervention to address heavy drinking in emergency care settings. | Multiple primary (same report) | Inadequate |  | Not registered | Not applicable | Not applicable |
| Morley, 2014 | To assess the impact of an opportunistic cognitive behavioral intervention package among adult outpatients with a substance use disorder and comorbid suicide risk. | Multiple primary (same report) | Inadequate |  | Not registered | Not applicable | Not applicable |
| Moss-Morris, 2013 | To test the effectiveness of a nurse-led cognitive behavioral therapy program to assist adjustment in the early stages of multiple sclerosis. | Multiple primary (same report) | Inadequate |  | Registered^*^ | ISRCTN91377356 | Inadequate |
| Naar-King, 2014 | To determine whether multisystemic therapy adapted for health care settings improved asthma management and health outcomes in high-risk African American adolescents with asthma. | Undefined | Inadequate |  | Registered^†^ | NCT00916240 | Inadequate |
| O'Brien, 2014 | To investigate whether family-focused therapy brought about greater improvements in family communication than enhanced care among individuals at clinically high risk for developing psychosis. | Multiple primary (same report) | Inadequate |  | Registered^†^ | NCT01907282 | Inadequate |
| Olthuis, 2014 | To test the efficacy of a telephone-delivered cognitive behavioral therapy intervention in reducing high anxiety sensitivity and its associated anxiety and depressive symptoms. | Primary | Adequate |  | Registered^*^ | NCT01194765 | Adequate/Consistent |
| Parsons, 2014 | To test a brief motivational interviewing intervention to reduce both risky sex and drug use among HIV-negative young gay and bisexual men. | Undefined | Inadequate |  | Registered^†^ | NCT01118416 | Inadequate |
| Pfeiffer, 2014 | To examine the effectiveness of a problem-solving intervention for stroke caregivers who provided care for at least 6 months and who experienced significant strain in their role. | Multiple primary (same report) | Inadequate |  | Registered^*^ | ISRCTN86289718 | Inadequate |
| Pfiffner, 2014 | To evaluate the efficacy of the Child Life and Attention Skills program, a behavioral psychosocial treatment integrated across home and school, for youth with attention-deficit/hyperactivity disorder-inattentive type. | Multiple primary (same report) | Inadequate |  | Registered^†^ | NCT00834821 | Inadequate |
| Price, 2014 | To explore a comprehensive predictive model for post-traumatic stress disorder to determine which variables were most indicative of reduced post-traumatic stress disorder symptoms for an early intervention or treatment as usual. | Multiple primary (same report) | Adequate |  | Registered^*^ | NCT00895518 | Inadequate |
| Reynolds, 2013 | To assess if cognitive behavioral therapy with high parental involvement was more effective than cognitive behavioral therapy with low parental involvement in reducing symptoms of obsessive-compulsive disorder. | Primary | Adequate |  | Not registered | Not applicable | Not applicable |
| Rogers, 2014 | To test a web-based trichotillomania self-help program to wait-list control on trichotillomania symptoms. | Undefined | Inadequate |  | Registered^†^ | NCT01118637 | Adequate/Inconsistent |
| Rogge, 2013 | To test three interventions for the primary prevention of marital distress and dissolution. | Undefined | Inadequate |  | Not registered | Not applicable | Not applicable |
| Rohde, 2014 | To evaluate 3 methods of integrating interventions for depression (Adolescent Coping With Depression Course) and substance use disorders (Functional Family Therapy) and examine treatment sequence effects on substance use and depression outcomes. | Multiple primary (same report) | Inadequate |  | Registered^†^ | NCT00680966 | Inadequate |
| Rohde, 2014 | To test whether a brief cognitive behavioral group and bibliotherapy prevention reduce major depressive disorder onset, depressive symptoms, and secondary outcomes relative to brochure controls in adolescents with self-reported depressive symptoms when school personnel recruit participants and deliver the intervention. | Multiple primary (same report) | Inadequate |  | Registered^†^ | NCT00904891 | Inadequate |
| Rose, 2014 | To evaluate the effectiveness of a friendship-building skills program--the Peer Interpersonal Relatedness program--in producing larger effects when used in conjunction with the Resourceful Adolescent Program. | Undefined | Inadequate |  | Not registered | Not applicable | Not applicable |
| Ruble, 2013 | To evaluate the Collaborative Model for Promoting Competence and Success teacher coaching intervention to a web-based program and placebo group for teachers of children with autism. | Multiple primary (same report) | Inadequate |  | Not registered | Not applicable | Not applicable |
| Schmidt, 2014 | To design and evaluate the efficacy of an anxiety sensitivity treatment more specifically focused on its cognitive component. | Multiple primary (same report) | Inadequate |  | Registered^†^ | NCT01947179 | Inadequate |
| Schneider, 2013 | To examine the efficacy of a disorder-specific treatment for separation anxiety compared to a general anxiety program for children suffering from separation anxiety disorder. | Multiple primary (same report) | Inadequate |  | Registered^*^ | NCT00255112 | Inadequate |
| Schumm, 2014 | To compare behavioral couples therapy with individually based therapy for women with alcohol use disorder. | Multiple primary (same report) | Inadequate |  | Not registered | Not applicable | Not applicable |
| Sergeant, 2014 | To compare an online positive psychology intervention to a control condition involving writing about daily activities on mental health outcomes. | Undefined | Inadequate |  | Not registered | Not applicable | Not applicable |
| Sikkema, 2013 | To examine whether Living in the Face of Trauma, a group intervention to address coping with HIV and childhood sexual abuse, significantly reduced traumatic stress over a 1-year follow-up period more than an attention-matched support group comparison intervention. | Primary | Adequate |  | Not registered | Not applicable | Not applicable |
| Simmons, 2013 | To test the efficacy of a dissonance-enhancing, web-based experiential intervention for increasing smoking cessation motivation and behavior. | Multiple primary (same report) | Inadequate |  | Not registered | Not applicable | Not applicable |
| Slesnick, 2013 | To examine the efficacy of 3 theoretically distinct interventions among substance-abusing runaway adolescents and to explore individual differences in trajectories of change. | Multiple primary (same report) | Inadequate |  | Not registered | Not applicable | Not applicable |
| Spoth, 2014 | To test whether a preventive intervention in middle school students compared to a life skills intervention and no intervention reduced substance use in late adolescence and early adulthood. | Undefined | Inadequate |  | Not registered | Not applicable | Not applicable |
| Stice, 2013 | To evaluate the effects of a prevention program targeting both eating disorders and obesity at 1- and 2-year follow-ups. | Multiple primary (same report) | Inadequate |  | Registered^†^ | NCT00433680 | Inadequate |
| Stiles-Shields, 2014 | To compare therapeutic alliance in clients receiving cognitive behavioral therapy for depression by telephone or face-to-face. | Secondary | Adequate |  | Registered^†^ | NCT00498706 | Excluded (secondary analysis) |
| Tellegen, 2014 | To evaluate the efficacy of Primary Care Stepping Stones Triple P, a brief individualized parenting program, in a sample of parents of children with autism spectrum disorder. | Undefined | Inadequate |  | Registered^*^ | ACTRN12612001196831 | Inadequate |
| ter Kuile, 2013 | To investigate the efficacy of therapist-aided exposure for lifelong vaginismus. | Primary | Adequate |  | Not registered | Not applicable | Not applicable |
| Vittengl, 2014 | To test cognitive therapy and fluoxetine versus pill placebo to prevent depression relapse among responders to acute-phase cognitive therapy. | Multiple primary (same report) | Inadequate |  | Registered^†^ | NCT00118404, NCT00183664, NCT00218764 | Inadequate |
| Wagner, 2014 | To examine criminal outcomes for siblings of serious and violent juvenile offenders who had participated on average 25.0 years earlier in a clinical trial of multisystemic therapy. | Secondary | Adequate |  | Not registered | Not applicable | Excluded (secondary analysis) |
| Wagner, 2014 | To evaluate the efficacy of a school-based guided self-change intervention compared to standard care for reducing substance use and aggressive behavior. | Multiple primary (same report) | Inadequate |  | Not registered | Not applicable | Not applicable |
| Webb Hooper, 2013 | To test the efficacy of placebo tailoring for smoking cessation and to examine the influence of cognitive processing style. | Multiple primary (same report) | Inadequate |  | Not registered | Not applicable | Not applicable |
| Weiss, 2013 | To conduct an independent evaluation of multisystemic therapy with non-court-referred adolescents with conduct problems. | Multiple primary (same report) | Inadequate |  | Not registered | Not applicable | Not applicable |
| White, 2013 | To test the effect of maintenance cognitive-behavioral therapy versus assessment only on sustained improvement and reduced relapse among responders to acute-phase treatment for panic disorder. | Multiple primary (same report) | Inadequate |  | Registered^†^ | NCT00000368 | Not applicable[^§^](http://en.wikipedia.org/wiki/Section_sign) |
| Williams, 2014 | To compare mindfulness-based cognitive therapy with both cognitive psychological education and treatment as usual in preventing relapse to major depressive disorder in people currently in remission following at least 3 previous episodes. | Primary | Adequate |  | Registered^*^ | ISRCTN97185214 | Inadequate |
| Williams, 2013 | To evaluate both the independent effects of a cognitive-bias modification protocol targeting imagery and interpretation bias and the combined effects of this intervention followed by an internet version versus a wait-list control in patients with a major depressive episode. | Multiple primary (same report) | Inadequate |  | Registered^*^ | ACTRN12611001221943, NCT01488058 | Inadequate |
| Wolchik, 2013 | To assess the effects of a preventive intervention for divorced families compared to a control condition. | Undefined | Inadequate |  | Registered^*^ | NCT01407120 | Inadequate |

ACTRN = Australian New Zealand Clinical Trials Registry; ISRCTN = International Standard Randomized Controlled Trial Number Register; NCT = ClinicalTrials.gov. ^*^ Registry name and number were reported in the article. ^†^ Registry name and number were not reported in the article. ^‡^In the last sentence of the Data Analytic Plan, the article mentions: “A Bonferroni correction of alpha = .025 was applied for significance for all outcomes to account for the use of multiple tests on the primary outcomes based on the number of tests within each category of outcome measure—self-report (PRCS, FNE-B) and behavioral avoidance task (length of speech and peak anxiety during speech)”. Thus, the trial has two primary outcome categories and, within each category, two primary variables. The authors correct for 2 primary outcomes within each category, but do not adjust for the fact that there are 2 primary categories, thus, a total of 4 primary outcomes. This was coded as multiple primary (without statistical correction). ^§^RCT ended before 2005.
